# Association between gut microbiota and peptic ulcer disease, particularly gastric ulcer and duodenal ulcer: a two-sample Mendelian randomization study

**DOI:** 10.3389/fmicb.2023.1277300

**Published:** 2024-01-12

**Authors:** Zhenhua Dong, Kai Yu, Yuchao Xin, Xulei Gao, Fan Bu, Dingliang Zhao, Donghui Ren, Ji Lu, Daguang Wang

**Affiliations:** ^1^Department of Gastric and Colorectal Surgery, The First Hospital of Jilin University Changchun, Jilin, China; ^2^Department of Urology, The First Hospital of Jilin University Changchun, Jilin, China; ^3^Second Department of Hepatobiliary and Pancreatic Surgery, The First Hospital of Jilin University Changchun, Jilin, China; ^4^Department of Plastic and Aesthetic Surgery, The First Hospital of Jilin University, Changchun, Jilin, China; ^5^Department of Second Urology, The First Hospital of Jilin University Changchun, Jilin, China

**Keywords:** gastric ulcer, duodenal ulcer, gut microbiota, causal inference, Mendelian randomization study, genetics

## Abstract

**Background:**

Recent an observational study has suggested a potential connection between gut microbiota (GM) and peptic ulcer diseases (PUDs), particularly gastric ulcer (GU) and duodenal ulcer (DU). However, the causal connection remains unsure.

**Methods:**

A two-sample Mendelian randomization (MR) is carried out to explore the connection between the GM and DU or GU. Data on the GM comes from the MiBioGend database, and GU or DU data are based on the FinnGen database. One group of single nucleotide polymorphisms (SNPs) (*P* < 5 × 10^−8^) are served as instrumental variables (IVs). To obtain a more comprehensive conclusion, the other SNPs (*P* < 1 × 10^−5^) are selected as IVs. Inverse variance weighting (IVW) is used to determine the causal relationship.

**Results:**

At the level of *P* < 1 × 10^−5^, the IVW analysis suggests that Clostridiaceae1, Butyriccoccus, and Peptcoccus have harmful effects on GU, while LachnospiraceaeUCG004 and MollicutesRF9 have beneficial effects on GU. Then, in the case of DU, the IVW analysis suggested that Lentisphaeria, Negativicutes, Clostridiaceae1, ClostridiumseMnsustricto1, ErysipelotrichaceaeUCG003, LachnospiraceaeNC2004group, Selenomonadale, Victivallales, and Lentisphaerae have harmful effects, while Catenibacterium, Escherichia.Shigella, LachnospiraceaeUCG008, and Sutterella have beneficial effects. When *P* < 5 × 10^−8^, IVW analysis suggests that GM has no significant influence on GU or DU.

**Conclusion:**

This two-sample MR indicates a causal relationship between GM and GU or DU.

## 1 Introduction

Peptic ulcer disease (PUD) is frequently encountered in clinical settings. PUD is characterized by the corrosion of the digestive tract lining, causing mucosal damage that extends into the submucosa. PUD usually occurs in the stomach or duodenum. Hence, the term PUD often refers to peptic gastric ulcer (GU) and duodenal ulcer (DU). The typical symptom of PUD mainly includes upper abdominal pain which occurs regularly, cyclically, seasonally, chronically. PUD may cause peptic bleeding, perforation, obstruction, and cancelation, significantly impacting people's physical health. Recently, the diagnosis of PUD depends on gastroscope and biopsy (Lanas and Chan, 2017). PUD is considered to be caused by several factors, mainly including Helicobacter pylori9 (Hp) infection and the unreasonable use of non-steroidal anti-inflammatory drugs (NSAIDs). Excessive drinking, smoking, and irregular diet are also the risk factors (Ramakrishnan and Salinas, 2007). The current treatment approach involves the use of proton pump inhibitors (PPIs) and antibiotics. Long-term use of antibiotics results in drug resistance of bacteria. Therefore, it is important to explore the etiology of PUD to provide patients with better treatment methods.

Due to improvement in gene sequencing technology, bacteria identification is widely applied to the biology study field. Some studies show that the overbalance of gut microbiota (GM) accompanies the entire process of PUD. Specifically, in patients who have PUD, the diversity and abundance of GM usually decreases (Chen et al., 2018). GM with the feature of large quantity and variety is essential for the digestive system (Eckburg et al., 2005). Nevertheless, it remains unclear as to whether there is a causal connection between GM and DU or GU.

Mendelian randomization is utilized to mine the database of genome-wide association study (GWAS), and to minimize greatly the impact of confounding factors. We often make use of Mendelian randomization (MR) to explore whether there are causal connections between exposure and outcomes. We choose single nucleotide polymorphisms (SNPs) that are significantly relevant to exposure as IVs to explore the causality. If they have causality, the outcome will be influenced by the selected IVs (Lawlor et al., 2008). In the current study, the two-sample MR was conducted to examine whether there is a causal connection between GM and GD or DU.

## 2 Materials and methods

### 2.1 Data source

SNPs of GM selected as instrument variables (IVs) were extracted from the MiBioGen database. The database includes 122,110 associated single nucleotide polymorphisms (SNPs) from 18,340 individuals. This is a large-scale GWAS that recruits 24 population-based cohorts and identifies 211 GMs. The European population occupied a significant proportion of the participants. The participants of the study come from the USA, Canada, Israel, South Korea, Germany, Denmark, the Netherlands, Belgium, Sweden, Finland, and the UK. These cohorts originating from single ancestries include European (16 cohorts, *N* = 13,266), Middle Eastern (one cohort, *N* = 481), East Asian (one cohort, *N* = 811), American Hispanic/Latin (one cohort, *N* = 1,097), and African American (one cohort, *N* = 114) populations. In addition, there are 4 cohorts consisting of 2,571 participants from multiple ancestries (Kurilshikov et al., 2021). Then, genetic summary statistics for GU, derived from FINNGEN, include 5,935 cases and 320,387 controls of the European ancestry. Genetic summary statistics for DU, also generated from FINNGEN, include 3,520 cases and 320,387 controls of the European ancestry. As the present study is based on public summary data, the study does not need additional ethics approval or consent to participate. The specific information of the data sources is shown in Table 1.

**Table 1:** Details of the genome-wide association studies and datasets used in our analyses.

**Exposure or outcome**	**Sample size**	**Links for data download**
Human gut microbiome	18,340 participants	https://mibiogen.gcc.rug.nl/
Gastric ulcer	5,935 cases, 32,0387 controls	https://storage.googleapis.com/finngen-public-data-r9/summary_stats/finngen_R9_K11_GULC.gz
Duodenal ulcer	3,520 cases, 32,0387 controls	https://storage.googleapis.com/finngen-public-data-r9/summary_stats/finngen_R9_K11_DULC.gz

### 2.2 Selection of instrumental variables

First, we removed 15 bacterial traits without a specific name, so 196 bacterial traits are left, including 9 phyla, 16 classes, 20 orders, 32 families, and 119 genera. Next, we selected the IVs at *P* < 1.0 × 10^−5^. For obtaining IVs from independent loci, we set the linkage disequilibrium (LD) threshold at R2 < 0.001 and the clumping distance of 10,000 kb in the EUR population reference using “Two Sample MR” packages of R software. SNPs that met these requirements were retained for clumping with 196 bacterial traits. A total of 2,699 independent SNPs were chosen to mate 196 bacterial traits. Additionally, we selected other IVs associated with GM at a stricter threshold (*P* < 5 × 10^−8^) when human GM is viewed as a whole, we screen these SNPs with same standards. Eventually, 16 independent SNPs were found. After extracting relevant information such as effect allele, β-value, standard error and *p*-value with each SNP, we calculated the proportion of variation explained (R2) and F-statistic to quantify the IV strength, with the following equation: R^2^ = 2 × MAF × (1 – MAF) × β^2^; F = R^2^ (n-k-1)/k(1-R), where “MAF” is the minor allele frequency of SNPs used as IVs, “n” is the sample size, and “k” is the number of IVs employed (Palmer et al., 2012). The above procedure of instrumental variable selection makes our research results more credible.

### 2.3 The assumptions of MR

To increase the credibility of the results, the MR should comply with three assumptions. First, IVs should influence exposure and the outcome without the influence of confounders. Second, the IVs should be greatly associated with exposure. We usually use F-statistic to assess the strength of the connection between IVs and exposure. If the connection is weak, with *F* < 10, we could eliminate these IVs. Third, IVs affect outcomes only through exposure which means that horizontal pleiotropy does not exist.

### 2.4 Mendelian randomization analysis

We explore the causal connection between exposure and outcomes by inverse variance weighted (IVW), MR-Egger, weighted median, and weighted mode, as well as simple mode. The nature of IVW is a mate analysis method. First, we calculate the causal effect ratio of each IV between the effect of IV on the outcome and exposure. Then, we make weighted regression for these ratios to evaluate the causal connection between GM on GU or DU. IVW assumes that the receptor of MR Egger is zero. If there is the horizontal pleiotropy, the result of IVW is unreliable (Choi et al., 2019). MR Egger improves IVW weakness, taking into account a certain level of pleiotropy. MR Egger is a method for quantization of the funnel plot. Even if all IVs are invalid, MR Egger still provides an unbiased result (Bowden et al., 2015). The weighted median method can reduce the occurrence of class 1 errors and draw a correct conclusion if < 50% IVs are invalid. Compared with MR Egger, the weighted median improves the accuracy of the results. Weighted mode and simple mode are Supplementary material. The weighted mode focus on IVs with similar causal estimates; if these IVs are valid, the result will be credible (Xiang et al., 2021). If the conclusions of these methods are inconsistent, we are more willing to rely on IVW under the assumption of no horizontal pleiotropy.

MR-Egger, Cochran's *Q*-test, and MR-PRESSO were used to test horizontal pleiotropy, heterogeneity, and outliers. We utilize the intercept of MR-Egger to check the existence of horizontal pleiotropy. If *P* > 0.05, it showed that there was no significant horizontal pleiotropy; hence, the outcome of IVW should be more reliable (Verbanck et al., 2018). MR-PRESSO is useful in checking the outliers and the stability of the results. Subsequently, Cochrane's Q test is used for testing heterogeneity among IVs. If IVs have significant heterogeneity, we should choose the random effect model; conversely, if IVs have no such heterogeneity, we tend to select the fixed effect model. All statistical analyses are performed using R software (version 4.2.3 and “Two Sample MR package”).

### 2.5 Linkage disequilibrium score regression

The MR result may be false positive if there is a shared genetic correlation between exposure and outcomes. Although we try our best to exclude SNPs related to outcomes as IVs, unrelated SNPs may influence the outcome through mediators, which is actually the meaning of pleiotropy and breaks the third premise of MR. Therefore, LDSC is utilized to calculate coinheritance by performing chi-squared statistics based on SNPs between exposure and outcomes. When the *p* > 0.05, it means that the shared genetic structure doesn't exist and the MR result can be more reliable. Meanwhile, when the *p* > 0.05, we must explore the shared SNPs by co-localization analysis of GWAS, and the MR result gets doubtful.

## 3 Results

### 3.1 Instrumental variable selection

For *P* < 1.0 × 10^−5^, we selected 2,699 dependent SNPs from a pool of 122,110 SNPs and extracted relevant information with these SNPs, such as beta exposure, standard error exposure exposure, *p*-value exposure and so on. Next, we harmonized these SNPs with SNPs of GU (outcome). Eventually, 2,432 SNPs was chosen by us. The specific information is shown in Supplementary Sheet 3. Similarly, we mated and merged 2,699 SNPs with SNPs of DU (outcome). Eventually, 2,471 SNPs were selected. The specific information is shown in Supplementary Sheet 4. While for *P* < 5.0 × 10^−8^, 16 SNPs were selected from 2,699 SNPs as IVs (GM and GU in Supplementary Sheet 9, GM and DU in Supplementary Sheet 10). Instrumental variables that possess strong predictive power can more accurately capture the characteristics of exposure factors. Employing robust instrumental variables in research can effectively mitigate the potential errors introduced by exposure when studying outcomes. All SNPs' F-statistic more than 10 indicated that there were not weak IVs in the results and this analysis is reliable (Supplementary Sheet 3).

### 3.2 Two-sample MR analysis

#### 3.2.1 Statistical threshold—P < 1.0 × 10^−5^

**Figure 1:**
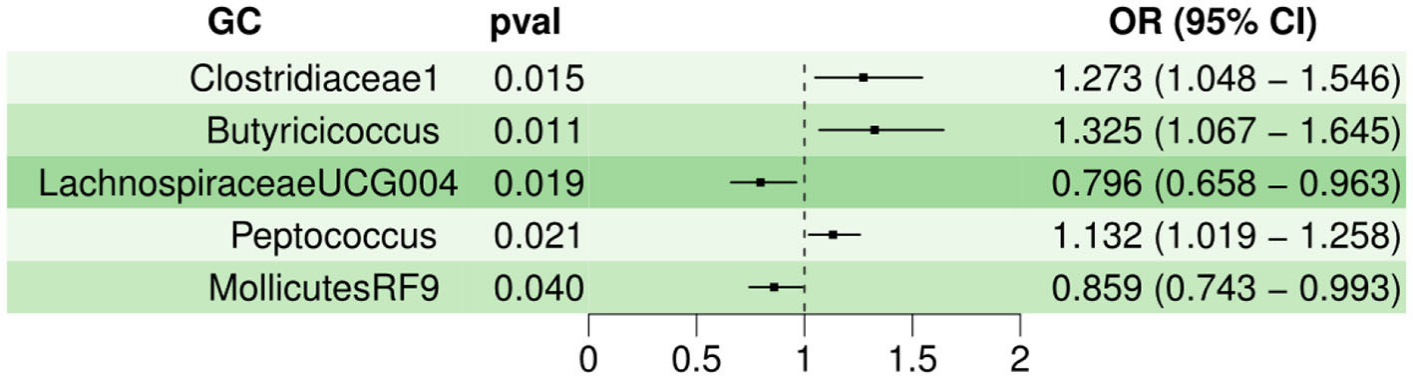
Forest plot of the causal effect of five types of bacteria on GU risk.

For various MR statistic methods, when *P* < 0.05, the results have statistical significance. The results of IVW analyses demonstrated that Clostridiaceae1 [OR = 1.273, 95% confidence interval (CI), 1.048–1.546, *P* < 0.05], Butyricicoccus (OR = 1.325, 95% CI, 1.067–1.645, *P* < 0.05), and Peptococcus (OR = 1.132, 95% CI, 1.019–1.258, *P* < 0.05) were positively correlated with the risk of GU. However, LachnospiraceaeUCG004 (OR = 0.796, 95% CI, 0.658–0.963, *P* < 0.05), and MollicutesRF9 (OR = 0.859, 95% CI, 0.743–0.993, *P* < 0.05) were negatively correlated with GU risk. In Cochran Q-test, five bacteria with a *p* > 0.05 indicate that there was no remarkable heterogeneity. MR-PRESSO was utilized to detect outliers and we set the distribution to 1000; however, we cannot find any outliers which will influence the results significantly. The detailed statistical results are shown in Supplementary Sheet 5. Characteristics of the genetic variants associated with five bacterial traits that have been identified to be associated with gastric ulcer (*P* < 1.00E-05) is shown in Supplementary Sheet 7. Furthermore, the horizontal pleiotropy between IVs and outcomes was assessed by the receptor of MR-Egger regression, Clostridiaceae1 (*P* = 0.56), Butyricicoccus (*P* = 0.81), LachnospiraceaeUCG004 (*P* = 0.95), Peptococcus (*P* = 0.02), and MollicutesRF9 (*P* = 0.47), indicating that no pleiotropy existed. The detailed statistical results of the 196 intestinal microbiomes are shown in Supplementary Sheet 1. Forest plot of the causal effect of five types of bacteria on GU risk is shown in Figure 1. Casual effects of TSMR analysis between gut microbiota and gastric ulcer (*P* < 1.00E-05) is shown in Supplementary Sheet 5.

**Figure 2:**
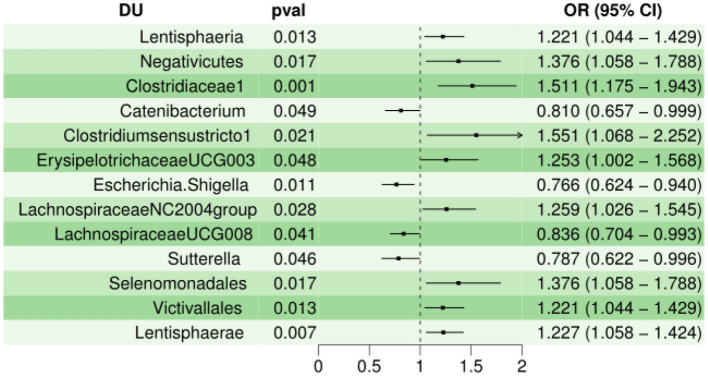
Forest plot of the causal effect of 13 types of bacteria on DU risk.

Next, the MR estimates of IVW indicated that Lentisphaeria (OR = 1.221, 95% CI, 1.044–1.429, *P* < 0.05), Negativicutes (OR = 1.376, 95% CI, 1.058–1.788, *P* < 0.05), Clostridiaceae1 (OR = 1.511, 95% CI, 1.18–1.94, *P* < 0.01), Clostridiumsensustricto1 (OR = 1.551, 95% CI, 1.068–2.252, *P* < 0.05), ErysipelotrichaceaeUCG003 (OR = 1.25,95% CI, 1.002–1.5, *P* < 0.05 ), LachnospiraceaeNC2004group (OR = 1.26, 95% CI, 1.026–1.545, *P* < 0.05), Selenomonadales (OR = 1.376, 95% CI, 1.058–1.788, *P* < 0.05), Victivallales (OR = 1.221, 95% CI, 1.044–1.429, *P* < 0.05), Lentisphaerae (OR = 1.227, 95% CI, 1.058–1.424, *P* < 0.01) were risk factors for DU, but Catenibacterium (OR = 0.810, 95% CI, 0.657–0.999, *P* < 0.05), Escherichia.Shigella (OR = 0.766, 95% CI, 0.624–0.940, *P* < 0.05), LachnospiraceaeUCG008 (OR = 0.836, 95% CI, 0.704–0.993, *P* < 0.05), Sutterella (OR = 0.787, 95% CI, 0.622–0.996, *P* < 0.05) served as protective factors for DU. In Cochran *Q*-test, 13 bacteria with *P* > 0.05 indicate that there was not remarkable heterogeneity. However, Clostridiumsensustricto1 (*P* < 0.05) may have potential heterogeneity. When MR-PRESSO was utilized to detect outliers and set the distribution to 1000, we cannot find any outliers. The horizontal pleiotropy between IVs and outcomes was assessed by the receptor of MR-Egger regression, Lentisphaeria (*P* = 0.90), Negativicutes (*P* = 0.94), Clostridiaceae1 (*P* = 0.67), Catenibacterium (*P* = 0.57), Clostridiumsensustricto1 (*P* = 0.65), ErysipelotrichaceaeUCG003 (*P* = 0.90), Escherichia.Shigella (*P* = 0.77), LachnospiraceaeNC2004group (*P* = 0.56), LachnospiraceaeUCG008 (*P* = 0.26), Sutterella (*P* = 0.63), Selenomonadales (*P* = 0.94), Victivallales (*P* = 0.90), and Lentisphaerae (*P* = 0.90), indicating that there was no pleiotropy. Characteristics of the genetic variants associated with 13 bacterial traits that have been identified to be associated with duodenal ulcer (*P* < 1.00E-05) is shown in Supplementary Sheet 8. The detailed statistical results of the 196 intestinal microbiomes are shown in Supplementary Sheet 2. Forest plot of the causal effect of 13 types of bacteria on DU risk is shown in Figure 2. Casual effects of TSMR analysis between gut microbiota and duodenal ulcer (*P* < 1.00E-05) is shown in Supplementary Sheet 6.

#### 3.2.2 Statistical threshold P < 5.0 × 10^−8^

**Figure 3:**
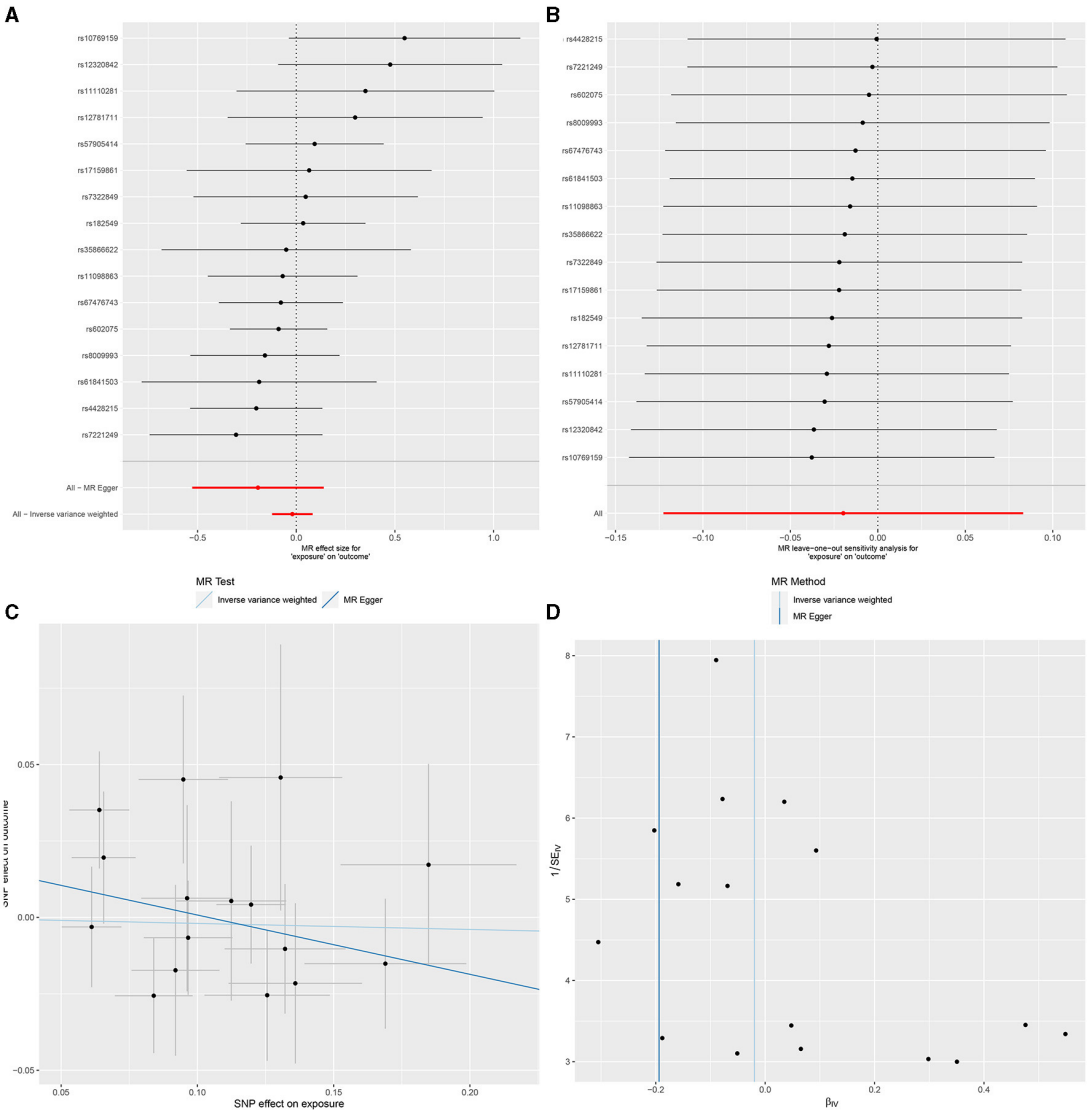
Gut microbiota as a whole and gastric ulcer. **(A)** Forest plot. **(B)** Sensitivity analysis plot. **(C)** Scatter plot. **(D)** Funnel plot.

**Figure 4:**
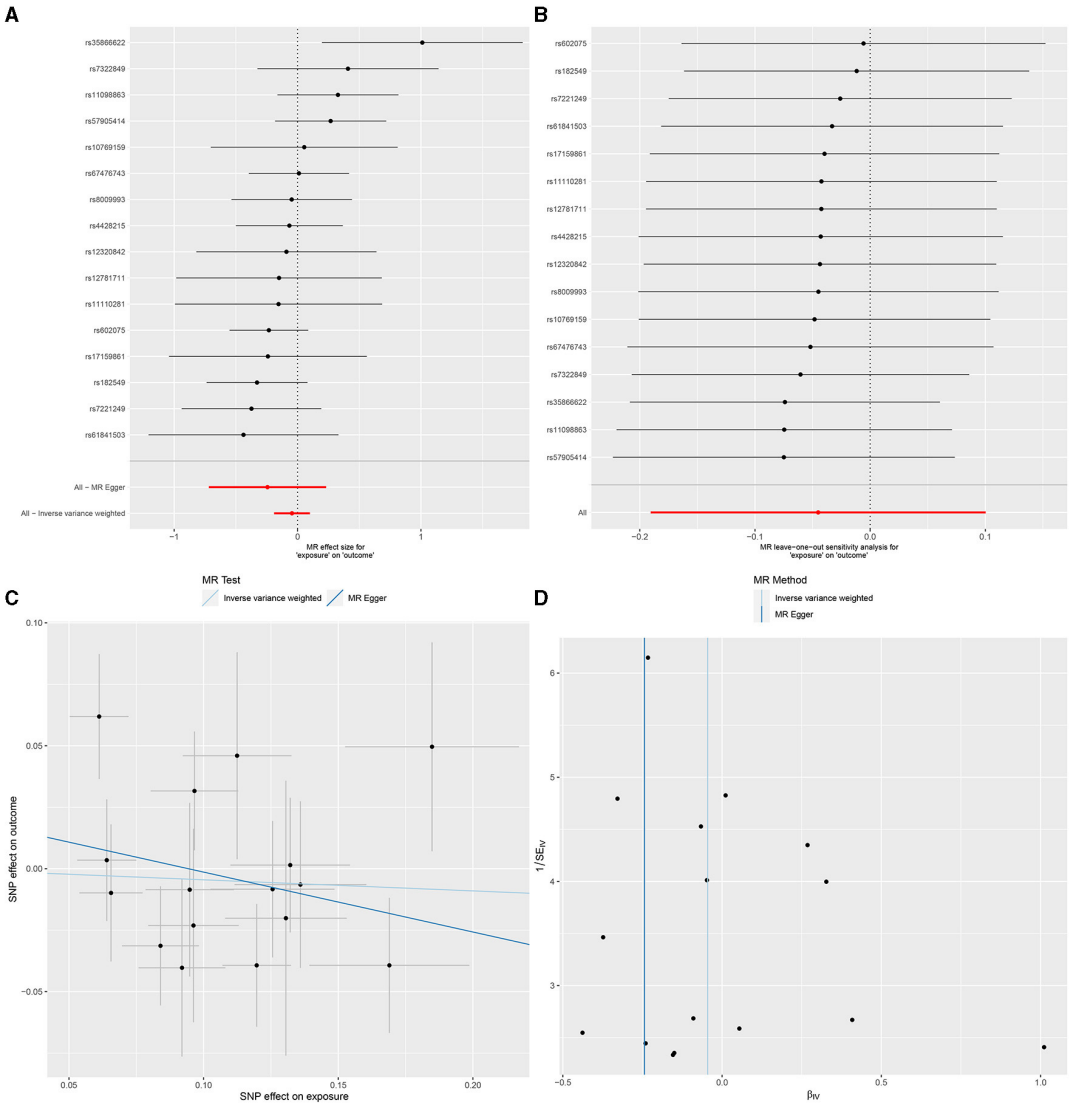
Gut microbiota as a whole and duodenal ulcer. **(A)** Forest plot. **(B)** Sensitivity analysis plot. **(C)** Scatter plot. **(D)** Funnel plot.

In IVW, GU and GM (OR = 0.980, 95% CI, 0.885–1.087, *P* > 0.05), DU and GM (OR = 0.956, 95% CI, 0.826–1.106, *P* > 0.05) showed that gut microbiome as a whole was not associated with GU or DU. The detailed statistical results could be found in GU in Supplementary Sheet 11 and DU in Supplementary Sheet 12. The forest plot (Figures 3A, 4A), sensitivity analysis (Figures 3B, 4B), scatter plot (Figures 3C, 4C), and funnel plot (Figures 3D, 4D) illustrating the causal effect of the bacteria as a whole on GU and DU risk are shown in Figures 3, 4.

### 3.3 Linkage disequilibrium score regression

Except the unit of DU and Clostridiaceae1 (Rg = −0.610, Se = 0.284, *P* < 0.05), there is little genetic correlation observed between GU and Clostridiaceae1 (Rg = −0.256, Se = 0.254, *P* > 0.05), Butyricicoccus (Rg = NA, Se = NA, *P* > 0.05), LachnospiraceaeUCG004 (Rg = 0.040, Se = 0.313, *P* > 0.05), Peptococcus (Rg = NA, Se = NA, *P* > 0.05), MollicutesRF9 (Rg = 5.646, Se = 3.376, *P* > 0.05), DU and Lentisphaeria (Rg = −3.054, Se = 4.406, *P* > 0.05), Negativicutes (Rg = 7.449, Se = 4.876, *P* > 0.05), Clostridiaceae1 (Rg = −0.610, Se = 0.284, *P* > 0.05), Catenibacterium (Rg = −0.927, Se = 1.260, *P* > 0.05), Clostridiumsensustricto1 (Rg = 10.250, Se = 6.120, *P* > 0.05), ErysipelotrichaceaeUCG003 (Rg = −1.087, Se = 5.231, *P* > 0.05), Escherichia.Shigella (Rg = 0.095, Se = 0.671, *P* > 0.05), LachnospiraceaeNC2004group (Rg = NA, Se = NA, *P* > 0.05), LachnospiraceaeUCG008 (Rg = NA, Se = NA, *P* > 0.05), Sutterella (Rg = 0.784, Se = 2.331, *P* > 0.05), Selenomonadales (Rg = 8.147, Se = 5.191, *P* > 0.05), Victivallales (Rg = −1.763, Se = 5.694, *P* > 0.05), and Lentisphaerae (Rg = −2.579, Se = 3.243, *P* > 0.05).

The value of Lambda usually indicates the bias of genetic structure and it varies from −0.364 (Clostridiumsensustricto1) to 0.117 (Lentisphaerae), which means the bias without existence. With the help of LDSC, we calculate the snp-heritage (proportion of snp explained for phenotype) of 18 bacterial traits; the value of h2 ranged from 0.003625 to 0.004078, which means that the contribution of heritage is considerable and detailed information is shown in Table 2.

**Table 2:** Genetic correlation estimates for gut microbiota and peptic ulcer disease by LDSC regression analysis.

**Exposure**	**Outcome**	**lambda**	**lambda_se**	**h2**	**h2_se**	**Rg**	**Rg_se**	**pval**
Clostridiaceae1	GU	−0.00062	0.0048	0.004078	0.001395	−0.25629	0.2538	0.31254
Butyricicoccus	GU	−0.00301	0.0047	0.004078	0.001395	NA	NA	0.92970
LachnospiraceaeUCG004	GU	0.00062	0.0046	0.004078	0.001395	0.03978	0.3130	0.89886
Peptococcus	GU	−0.07342	0.0670	0.004078	0.001395	NA	NA	0.38356
MollicutesRF9	GU	−0.08884	0.1375	0.004078	0.001395	5.64576	3.3763	0.09449
Lentisphaeria	DU	0.10533	0.1503	0.003625	0.001497	−3.05392	4.4057	0.48820
Negativicutes	DU	−0.06414	0.1255	0.003625	0.001497	7.44893	4.8761	0.12660
Clostridiaceae1	DU	0.00243	0.0049	0.003625	0.001497	−0.60963	0.2841	0.03191
Catenibacterium	DU	0.00331	0.0058	0.003625	0.001497	−0.92712	1.2602	0.46191
Clostridiumsensustricto1	DU	−0.36415	0.1324	0.003625	0.001497	10.25015	6.1200	0.09396
ErysipelotrichaceaeUCG003	DU	−0.06007	0.1451	0.003625	0.001497	−1.08657	5.2313	0.83546
Escherichia.Shigella	DU	0.00267	0.0053	0.003625	0.001497	0.09492	0.6705	0.88741
LachnospiraceaeNC2004group	DU	−0.06398	0.1424	0.003625	0.001497	NA	NA	0.58161
LachnospiraceaeUCG008	DU	−0.03769	0.1171	0.003625	0.001497	NA	NA	0.76037
Sutterella	DU	−0.03237	0.1274	0.003625	0.001497	0.78447	2.3312	0.73649
Selenomonadales	DU	−0.07945	0.1297	0.003625	0.001497	8.14660	5.1908	0.11655
Victivallales	DU	0.03513	0.1921	0.003625	0.001497	−1.76343	5.6943	0.75680
Lentisphaerae	DU	0.11682	0.1596	0.003625	0.001497	−2.57883	3.2430	0.42650

## 4 Discussion

Due to the presence of strong acidic substances, the stomach is considered as an organ without bacteria (Gillespie, 1981). However, the discovery of changes human cognition. What is most important is that Hp have a relative relationship with chronic gastritis and PUD (Xu et al., 2022). With the development of mass spectrometry biotyping analysis and 16S rRNA high-throughput sequencing analysis, an increasing number of bacteria are discovered, which constitute the stomach microbial system. For example, a study about molecular analysis of the bacterial microbiota in the human has identified many ribosomal DNA sequences from a wealth of bacteria including Caulobacter, Actinobacillus, Corynebacterium, Rothia, Gemella, Leptotrichia, Porphyromonas, Capnocytophaga, TM7, Flexistipes, and Deinococcus (Bik et al., 2006).

Nonetheless, we know little about the stomach microbial system, with even less knowledge about the differences in microbial composition between GU and DU. However, it is undeniable that many scientific studies have made efforts to achieve this. For instance, depending on 16S rRNA high-throughput sequencing analysis, they found that Firmicutes and Streptococcus were enriched in the stomach of gastritis patients who did not have Hp infection (Li et al., 2009). Furthermore, based on spectrometry biotyping analysis, Streptococcus, Neisseria, Rothia, and Staphylococcus are found to be dominant species in the stomachs of patients with Hp infection (Hu et al., 2012). In another study, it was observed that, for DU patients, not only the proportion of infecting Hp is significantly lower compared to GU patients but also the proportion of Bacteroides and Streptomyces is significantly higher than those with GU (Chen et al., 2018).

In comparison with the above study outcomes, we possess a large sample size based on the FINNEN public database. Qualified SNPs are selected as IVs to eliminate greatly confounding factors. This enables us to arrive at a more comprehensive conclusion. A two-sample Mendelian randomization (MR) is conducted to conclude that five bacteria are related to GU, where two are protective factors and three are risk factors. In the case of DU, 13 bacteria are associated, where four are protective factors and nine are risk factors. As a result, we point out that the diversity and abundance of microbiota in the mucosal tissue of DU patients are higher than those of GU patients. This view is supported by previous studies (Chen et al., 2018). However, the detailed mechanisms of bacteria remains largely unknown. Some bacteria, such as LachnospiraceaeUCG004, MollicutesRF9, Catenibacterium, Escherichia, Shigella, LachnospiraceaeUCG008, and Sutterella, may protect the patients with PUD through the following ways. First, the cytokine reaction triggered by Hp may could be dampened by them. Second, Hp infection may cause the deficiency ofstomach acid; however, some bacteria secreting lactic acid may improve this situation. Third, the harmful bacteria are difficult to adhere to epithelial cells because host surface receptors have been occupied by protective bacteria. Last, probiotics may have the potential to directly kill harmful bacteria (Boltin, 2016).

PUD mainly results from infection and the use of NSAIDs. Therefore, eradicating Hp through the use of antibiotics, PPIs, and reasonable use of NSAIDs are our current treatment strategy. However, in some areas, the problem of macrolide antibiotic resistance has been increasingly severe, to the extent that clarithromycin triple therapy may no longer be the first choice (Guevara and Cogdill, 2020). A study suggests that PUD needs a long-term treatment. Compared with untreated patients, those accepting continuous acid suppression therapy have lower likelihood of recurrence and are rarely featured by serious complications (Dobrilla et al., 1993). However, the long-term use of PPI could raise the risk of fractures, interact with antiplatelet medications, contribute to chronic kidney disease, increase susceptibility to difficile infection, and potentially be associated with dementia, as well as lead to deficiencies in magnesium, calcium, and vitamin B12 micronutrients (McConaghy et al., 2023). Thus, we need to add probiotics to increase the efficiency of treatment and decrease adverse reactions (Homan and Orel, 2015). For PUD, people gradually accept using Lactobacillus, Bifidobacterium, and Saccharomyces as probiotics (Boltin, 2016). The result of MR may provide a new direction for people to explore more probiotics and guide people to use antibiotics reasonably to some extent. By exploring the causal relationship between intestinal flora and ulcerative diseases of the digestive system, we can prevent and treat diseases in their early stages through the culture of fecal bacteriology in clinical practice, which can become an effective prediction tool for diseases.

However, we have to admit several limitations in our analysis. First, we did not evaluate the associations between GM and PUD when samples come from different age groups due to a lack of relevant GWASs. Second, we cannot evaluate potential non-linear links because this analysis relies on a public database. Third, sample overlap in the GWASs of GM and PUD (FINNEN) are likely to influence the causal estimates and inflate Type 1 error rates in the primary analysis (Burgess et al., 2016). Finally, the generalizability of this study may be limited because of participants mainly consist of Europeans.

## 5 Conclusion

Peptic ulcer diseases are always accompanied by changes in the gut microbiota.

To some extent, changes in the GM causes the occurrence of PUD; thus, effective intestinal microbiota detection can predict the occurrence of intestinal disease in time, and more effective intervention can be carried out in the early stage of the disease. For those who have PUD, adding reasonable probiotics according to the result of GM detection may increase the efficiency of treatment.

## Data availability statement

The original contributions presented in the study are included in the article/Supplementary material, further inquiries can be directed to the corresponding author.

## Ethics statement

Ethical approval was not required for the study involving humans in accordance with the local legislation and institutional requirements. Written informed consent to participate in this study was not required from the participants or the participants' legal guardians/next of kin in accordance with the national legislation and the institutional requirements. The manuscript research not on animals that do not require ethical approval for our study.

## Author contributions

KY: Writing – review & editing. ZD: Writing – original draft. YX: Conceptualization, Formal analysis, Investigation, Writing – original draft. XG: Methodology, Resources, Supervision, Writing – review & editing. FB: Data curation, Writing – review & editing. DZ: Supervision, Writing – review & editing. DR: Methodology, Writing – review & editing. JL: Funding acquisition, Project administration, Supervision, Writing – original draft. DW: Project administration, Software, Validation, Writing – review & editing.
